# Injectable electroconductive Prussian blue nanofiber-PVA hydrogel modulates the wound microenvironment to promote diabetic wound healing

**DOI:** 10.3389/fbioe.2026.1748784

**Published:** 2026-02-16

**Authors:** Dian-Qing Wang, Jin Huang, Sheng Chang

**Affiliations:** 1 Department of Orthopedics, The First Affiliated Hospital of Jinzhou Medical University, Jinzhou, Liaoning, China; 2 Key Surgical Laboratory of Educational Administration of Liaoning Province, The First Affiliated Hospital of Jinzhou Medical University, Jinzhou, Liaoning, China; 3 Department of Stomatology, The First Affiliated Hospital of Jinzhou Medical University, Jinzhou, Liaoning, China

**Keywords:** diabetic wound healing, electroactive biomaterial, hydrogel, injectable hydrogel, Prussian blue nanofiber

## Abstract

Chronic diabetic wounds exhibit persistent oxidative stress, prolonged inflammation, impaired angiogenesis, and a disrupted bioelectric microenvironment that hinders re-epithelialization. Here, we develop an injectable Prussian Blue nanofiber-PVA hydrogel (PBM.PVA gel) with electroconductive and immunomodulatory features for accelerated diabetic wound repair. Electrospun PBM nanofibers were uniformly embedded within a physically cross linked PVA matrix, producing a flexible and adhesive composite with stable conductivity. *In vitro*, PBM. PVA gel showed excellent cytocompatibility, reduced pro-inflammatory cytokines (IL-6, TNF-α, CD86), and enhanced pro-regenerative markers (CD206, CD31). In streptozotocin-induced diabetic mice, the hydrogel significantly accelerated wound closure, reduced inflammatory infiltration, and promoted collagen deposition with increased CD31-positive staining. While PBM has been reported to possess redox-regulatory potential, ROS levels and endogenous wound electrical fields were not directly quantified in this study; therefore, mechanistic interpretations are described as plausible and require further validation. Together, PBM. PVA gel provides a multifunctional dressing that supports a favorable wound microenvironment and improves healing outcomes in diabetic wounds.

## 1 Introduction

Chronic diabetic wounds represent a severe clinical complication characterized by persistent inflammation, elevated oxidative stress, and impaired angiogenesis (Guan et al., 2021; Tan et al., 2023; Xiang et al., 2024). The hyperglycemic microenvironment suppresses fibroblast migration, delays collagen deposition, and disrupts redox homeostasis, resulting in poor wound closure. Moreover, diabetes-induced metabolic dysregulation further aggravates wound-healing disorders by impairing cellular responses and tissue regeneration (Xu et al., 2016; Hu et al., 2021; Liu et al., 2023). Hydrogel-based dressings, with their hydrophilic networks and tissue-like elasticity, have gained attention for maintaining a moist environment, absorbing exudates, and supporting cell infiltration (Huang et al., 2022; Fan et al., 2024; Mo et al., 2025). Beyond conventional hydrogel systems, emerging bioactive scaffolds have demonstrated the ability to modulate local immune responses and regulate cellular behavior in chronic wound settings (Zhu et al., 2023; Liu et al., 2025). However, few hydrogel systems can simultaneously regulate oxidative stress and chronic inflammation in the diabetic wound microenvironment, which remains a major challenge in the treatment of chronic diabetic wounds. Among various hydrogels, polyvinyl alcohol (PVA) exhibits high biocompatibility, flexibility, and chemical stability. Its limited intrinsic bioactivity restricts its therapeutic efficacy in complex pathological wounds (Liang et al., 2021; Huang et al., 2023; Wang et al., 2023). Prussian Blue nanoparticles (PB NPs) have been widely reported to possess catalytic and redox-regulatory potential, and have shown promise in modulating inflammatory responses in wound-related studies (Busquets and Estelrich, 2020; Xu et al., 2022; Han et al., 2025; Lu et al., 2025; Xie et al., 2025). Nevertheless, PB NPs are prone to aggregation and loss of activity when directly incorporated into polymeric matrices.

Here, we designed a hybrid material by integrating electrospun Prussian Blue nanofiber membranes (PBM) into a PVA hydrogel to construct an injectable composite (PBM.PVA gel). The nanofibrous PBM component provides structural reinforcement and electroactive features, while the PVA matrix imparts injectability and tissue adhesion. We hypothesized that this composite hydrogel could provide mechanical integrity, biocompatibility, and biochemical cues to modulate the inflammatory microenvironment and support angiogenic responses (Zhang et al., 2025; Zhu et al., 2025; Zhou et al., 2026). In addition to biochemical regulation, diabetic wounds suffer from a severely disrupted endogenous bioelectric field, which normally guides keratinocyte migration and vascular activation. Embedding electroactive PBM nanofibers into a PVA hydrogel is therefore expected to facilitate the transmission of endogenous bioelectric cues, potentially supporting re-epithelialization and neovascularization without external electrical stimulation (Zhao et al., 2023; Zhang and Liu, 2025).

To validate this hypothesis, we systematically investigated (1) the structural and mechanical characteristics of PBM. PVA gel, (2) its adhesion, injectability, and hemocompatibility, (3) its cytocompatibility and immunomodulatory performance in RAW264.7 and HUVEC cells, and (4) its wound healing efficacy in a murine full-thickness diabetic wound model.

## 2 Materials

### 2.1 Experimental section

#### 2.1.1 Preparation of Prussian blue nanofiber membranes (PBM)

Poly (lactic acid) (PLA, Mw = 100,000), polyvinyl alcohol (PVA, Mw = 89,000–98,000, 99% hydrolyzed), Prussian Blue nanoparticles (PB NPs), and hexafluoroisopropanol (HFIP, 99%) were purchased from Sigma-Aldrich and used without further purification. All cell culture media and reagents were obtained from Gibco unless otherwise stated. Deionized water (18.2 MΩ cm) was used for all aqueous preparations. PLA (20% w/v) was dissolved in HFIP and stirred for 12 h at room temperature. PB nanoparticles were added at concentrations of 0, 0.25, 0.5, 1.0, and 2.0 wt% (relative to PLA weight) and ultrasonicated for 30 min to achieve homogeneous dispersion. The electrospinning process was conducted at a voltage of 20 kV, a tip-to-collector distance of 18 cm, and a feeding rate of 0.6 mL/h under ambient conditions. The as-spun membranes were vacuum-dried for 72 h to remove residual solvents. To obtain PBM fiber fragments suitable for hydrogel fabrication, the electrospun PBM membranes were cut into small pieces (approximately 3–5 mm in size). A total of 20 mg of PBM membrane was dispersed in 10 mL of deionized water and then shredded using a high-speed homogenizer (Ultra-Turrax T25, IKA, Germany) at 10,000 rpm for 3 min. Homogenization was performed under ambient conditions, and the resulting fiber suspension was used directly without filtration, centrifugation, or further size selection.

Scanning electron microscopy (SEM) images were acquired using a field-emission SEM (SU8010, Hitachi, Japan) operated at an accelerating voltage of 5 kV. Images were collected at representative magnifications using secondary-electron mode with a typical working distance of 8–10 mm. Prior to imaging, samples were sputter-coated with a thin Au layer (approximately 5–10 nm) to minimize charging. Fluorescence images were obtained using an inverted fluorescence microscope (IX73, Olympus, Japan) with the same objective lens and fixed exposure settings for all groups within each staining batch to enable quantitative comparison. Images were exported in the same format and analyzed using ImageJ under identical thresholding/measurement parametersunder identical acquisition settings for all experimental groups. Image processing and quantitative analysis were performed using ImageJ software (National Institutes of Health, United States). Based on preliminary characterization, the sample containing 0.5 wt% PB was selected as the optimized PBM for subsequent experiments.

### 2.2 Fabrication of injectable PBM.PVA hydrogel (PBM.PVA gel)

PVA solution (10% w/v) was prepared by dissolving PVA in deionized water at 90 °C under constant stirring until fully transparent. The electrospun PBM was shredded into small fibers and uniformly dispersed in PVA solution (mass ratio 1:4). This ratio was empirically determined based on preliminary experiments aimed at achieving a balance between injectability, mechanical integrity, and homogeneous fiber dispersion. The resulting mixture was subjected to three freeze-thaw cycles to induce physical crosslinking, with each cycle consisting of freezing at −20 °C for 8 h followed by thawing at 25 °C for 4 h. The mixture was cast into sealed molds (typical volume: 2 mL per sample) prior to freeze-thaw processing to minimize dehydration. For each cycle, samples were frozen statically at −20 °C and then thawed at 25 °C under static conditions. After three cycles, the formed hydrogels were stored in sealed containers at 4 °C and used within 48 h unless otherwise stated. After completion of the freeze-thaw process, an injectable PBM. PVA hydrogel was obtained. The same procedure without the addition of PBM fibers was used to prepare the pure PVA hydrogel as a control.

### 2.3 Characterization

The surface morphology of PBM, PVA, and PBM. PVA gels was observed using field-emission scanning electron microscopy (FE-SEM, Hitachi S4800). Fiber diameter of PBM was quantified from SEM images by measuring the shortest distance across individual fibers using ImageJ. At least 60 fibers were randomly selected from three representative SEM fields per sample. For hydrogels, pore size was quantified from SEM images by measuring the equivalent pore diameter from randomly selected pores (n ≥ 60 per group), and the results were presented as size-distribution histograms. Fourier transform infrared (FTIR) spectra were recorded using a Nicolet iS50 spectrometer to confirm chemical composition. A detailed FTIR peak assignment table (Supplementary Table S1) has now been added to the Supplementary Material, listing characteristic peaks of PBM and PBM. PVA hydrogel with corresponding functional groups. The elastic modulus was determined using a dynamic mechanical analyzer (DMA Q800, TA Instruments) at 1 Hz frequency. Rheological measurements were performed using a rotational rheometer (MCR 302, Anton Paar, Austria) equipped with a 20 mm diameter parallel-plate geometry. Measurements were conducted using a temperature-controlled stage set to 37 °C, and samples were equilibrated at 37 °C for 5–10 min prior to testing. Frequency sweep tests were conducted at 37 °C over an angular frequency range of 0.1–100 rad s^-1^ under a constant strain of 1%, which was within the linear viscoelastic region. The storage modulus (G′) and loss modulus (G″) were recorded to evaluate the viscoelastic stability of the hydrogels. Shear viscosity measurements were performed using the same rheometer (MCR 302, Anton Paar, Austria) at 37 °C with a 20 mm parallel-plate geometry. The viscosity of the PBM. PVA hydrogel was recorded as a function of shear rate ranging from 0.1 to 1,000 s^-1^ to evaluate shear-thinning behavior relevant to injectability. Swelling ratios were calculated as (Ws - Wd)/Wd × 100%, where Wd and Ws represent the dry and swollen weights, respectively, measured at various time intervals (0–48 h).

### 2.4 Hemocompatibility and adhesion tests

Hemolysis assays were conducted using diluted mouse blood (5%, v/v) to obtain a measurable and linear hemoglobin signal for spectrophotometric quantification. Briefly, freshly collected mouse blood was mixed with physiological saline to prepare a 5% (v/v) blood suspension. Samples were incubated with 1.5 mL of the diluted blood at 37 °C for 1 h, followed by centrifugation at 1,500 rpm for 5 min. The absorbance of the supernatant was recorded at 540 nm. Undiluted whole blood was not used for hemolysis quantification because high cell density and rapid clotting/aggregation can compromise optical measurement accuracy and reproducibility; therefore, diluted blood is commonly adopted for standardized hemolysis testing of biomaterials. Adhesion strength was evaluated using a lap-shear test on various substrates including aluminum alloy, iron, glass, ceramic, PE, wood, rubber, beef, and shrimp, under both dry and wet conditions. Lap-shear tests were performed using a universal testing machine (Instron 5,944, Instron Corporation, United States) at a constant crosshead speed of 10 mm min^-1^ with a defined overlap area of 10 × 10 mm^2^. The lap-shear strength (kPa) was calculated by dividing the maximum load at failure by the overlap area. All measurements were performed in triplicate (n = 5).

### 2.5 *In vitro* cytotoxicity assay at different hydrogel concentrations

To evaluate the concentration-dependent cytocompatibility of the final PBM. PVA hydrogel formulation, extract solutions were prepared according to ISO 10993–12. Briefly, the hydrogels were incubated in complete culture medium at 37 °C for 24 h, and the resulting extracts were serially diluted with fresh medium to obtain 25%, 50%, 75%, and 100% extract concentrations. RAW264.7 macrophages and HUVECs were cultured with the different extract dilutions for 24 h, and cell viability was assessed using the CCK-8 assay according to the manufacturer’s instructions. Absorbance was measured at 450 nm using a microplate reader.

### 2.6 Immunofluorescence staining and Western blot analysis

RAW264.7 cells were stimulated with LPS (1 μg/mL) for 12 h and treated with each sample group (Control, PVA, PBM, PBM. PVA gel). After 48 h, cells were fixed and stained for IL-6, TNF-α, CD86, CD206, and CD31. Nuclei were counterstained with DAPI. Fluorescence intensity was quantified using ImageJ. RAW264.7 macrophages were lysed using RIPA buffer containing protease and phosphatase inhibitors. Total protein concentrations were determined using a BCA protein assay kit. Equal amounts of protein (20–30 μg) were separated by SDS-PAGE and transferred onto PVDF membranes. After blocking with 5% non-fat milk, membranes were incubated overnight at 4 °C with primary antibodies against iNOS, Arg-1, IL-10, and tubulin (loading control). The membranes were then incubated with HRP-conjugated secondary antibodies and visualized using enhanced chemiluminescence (ECL). Band intensities were quantified using ImageJ software.

### 2.7 *In Vivo* diabetic wound healing model

All animal procedures were approved by the Institutional Animal Ethics Committee of Jinzhou Medical University (approval number: 240,128–5). Mice were randomly assigned to treatment groups (PBS control, PVA, PBM, PBM. PVA; n = 15 per group) using a random-number method. Male C57BL/6 mice (8 weeks old) were rendered diabetic by intraperitoneal injection of streptozotocin (STZ, 50 mg/kg) for five consecutive days. Mice with fasting blood glucose >16.7 mmol/L for two consecutive measurements were considered diabetic and used for subsequent experiments. The fasting blood glucose levels of individual mice in all four groups (n = 15 per group) were monitored at baseline (before STZ administration), and at 1 week and 4 weeks after STZ administration. As shown in Supplementary Table S2, all STZ-treated mice exhibited fasting blood glucose levels exceeding 16.7 mmol/L for two consecutive measurements, confirming successful establishment of the diabetic model prior to wound creation. After 4 weeks of diabetes induction, full-thickness skin defects (10 mm) were created on the dorsal surface. The wounds were randomly treated with PBS (Control), PVA hydrogel, PBM membrane, or PBM. PVA gel. These groups were selected as platform-matched controls to decouple the effects of the PVA matrix (PVA vs. Control), PB-containing fibrous network alone (PBM), and the composite integration of PBM within the hydrogel (PBM.PVA).

Wound areas were photographed on days 0, 3, 7, and 14. Wound closure was calculated as:

$$\text{Wound closure }\left(\%\right)=\left(A_{0}-A_{t}\right)/A_{0}\times 100$$

where A_0_ and A_t_ denote the wound area at day 0 and time t.

### 2.8 Histological and immunofluorescence analysis

Excised wound tissues were fixed, paraffin-embedded, and sectioned for hematoxylin and eosin (H&E) staining. Immunofluorescence analysis of IL-6, TNF-α, CD86, CD206, and CD31 was performed on days 3, 7, and 14 to assess inflammation and angiogenesis.

### 2.9 Statistical analysis

All experiments were conducted in triplicate. Data are presented as mean ± standard deviation (SD). Statistical analysis was performed using one-way ANOVA with Tukey’s post hoc test. Significance was set at p < 0.05.

## 3 Results and discussion

**FIGURE 1:**
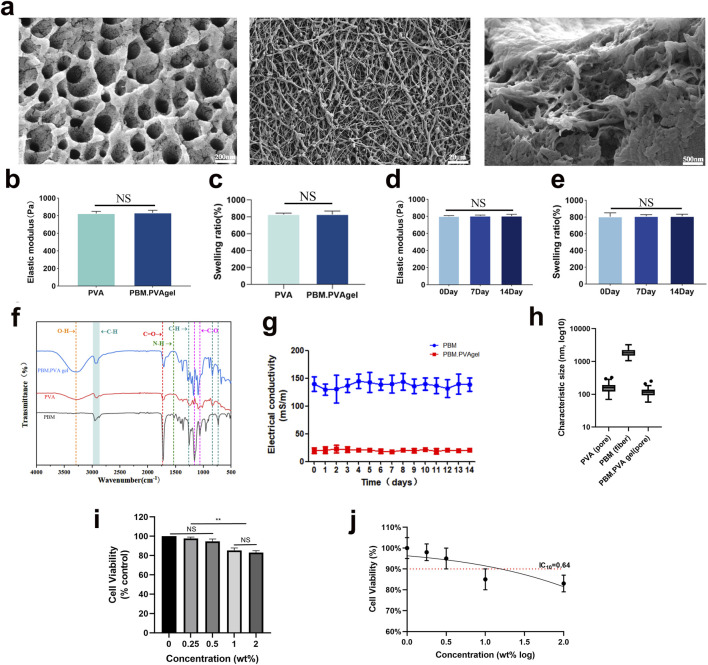
Structural, mechanical, and functional characterization of the PBM. PVA hydrogel. **(a)** Representative SEM images showing the porous PVA hydrogel network, fibrous PBM nanofiber membrane, and porous PBM. PVA composite hydrogel containing embedded PBM fibers. **(b, c)** Elastic modulus and swelling ratio of PVA and PBM. PVA hydrogels. **(d, e)** Mechanical and swelling stability after 0, 7, and 14 days of PBS immersion. **(f)** FTIR spectra confirming integration of PBM into the PVA network. **(g)** Electrical conductivity of PBM membrane and PBM. PVA hydrogel over 14 days **(h)** Quantitative characteristic size distributions of PVA hydrogel, PBM nanofiber membrane, and PBM. PVA composite hydrogel derived from SEM images. **(i)** CCK-8 assay evaluating concentration-dependent cell viability. **(j)** Determination of the IC10 value (0.64 wt%) derived from the CCK-8 viability analysis. Data are presented as mean ± SD (n = 3). Quantitative fiber diameter analysis of PBM prior to embedding is provided in Supplementary Figure S1.

The structural and physicochemical characteristics of the PBM. PVA hydrogel were investigated to evaluate its feasibility as a multifunctional dressing for chronic diabetic wounds. Figure 1a shows that the pristine PVA hydrogel exhibits an interconnected porous microstructure, whereas the electrospun PBM nanofibers form a continuous and uniform fibrous network (Zheng et al., 2025). After incorporation into the PVA matrix, PBM nanofibers were homogeneously distributed without visible aggregation, confirming excellent interfacial compatibility and the successful formation of a composite network. Due to the porous nature of the PBM. PVA composite hydrogel, quantitative structural analysis after embedding focused on pore size distribution rather than fiber diameter, whereas fiber diameter was quantified for PBM prior to embedding (Figure 1h; Supplementary Figure S1). These two metrics describe different dominant microstructural features (fiber diameter for PBM versus pore size for hydrogels) and are therefore reported separately to avoid misleading direct numerical comparison. Mechanical analysis further revealed that although the introduction of PBM slightly reduced the elastic modulus (Figure 1b), the PBM. PVA hydrogel preserved appropriate flexibility to accommodate dynamic wound deformation. As shown in Figure 1c, the swelling ratios of PVA and PBM. PVA hydrogels were nearly identical, and both mechanical strength and swelling ratios remained stable over 14 days of PBS immersion (Figures 1d,e), indicating good long-term structural robustness.

**FIGURE 2:**
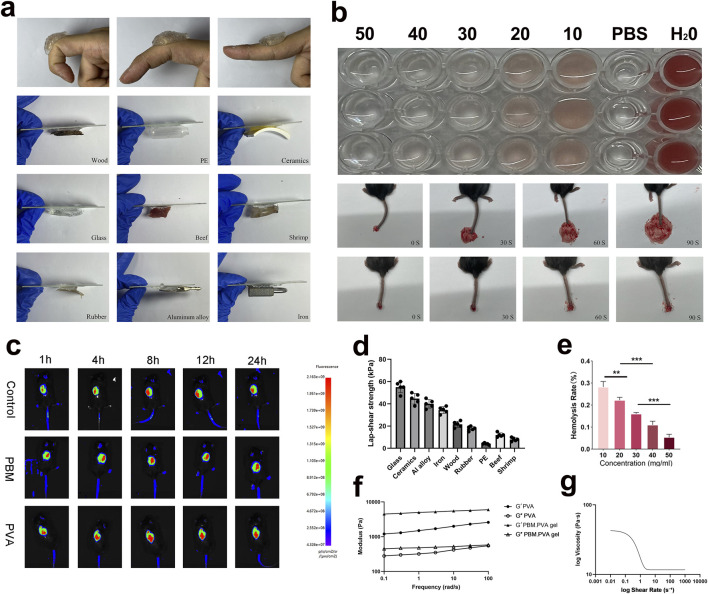
Adhesion, hemocompatibility, and injectability of PBM. PVA hydrogels. **(a)** Adhesion performance on human skin and various substrates under dry/wet conditions. **(b)** Hemolysis assay confirming <1% hemolysis, diluted mouse blood (5%, v/v) was used for hemolysis quantification to ensure reliable spectrophotometric measurement at 540 nm. **(c)** Fluorescence imaging of FITC-labeled hydrogel showing injectability and retention *in vivo*. **(d)** Lap-shear strength of PBM. PVA hydrogel on different substrates, including glass, ceramic, aluminum, wood, rubber, polyethylene (PE), beef tissue, and shrimp tissue. **(e)** Hemostatic performance of PBM. PVA gel evaluated by blood clotting index. **(f)** Frequency-dependent storage modulus (G′) and loss modulus (G″) of PVA and PBM. PVA gel. **(g)** Shear rate-dependent viscosity of PBM. PVA gel. Data are presented as mean ± SD (n = 3).

Chemical composition was verified by FTIR spectroscopy (Figure 1f), where characteristic O-H, C-H, C=O, and C≡N vibrations confirmed the coexistence of PVA and PBM within the hybrid network. Particularly noteworthy is that the PBM nanofibers displayed high intrinsic electrical conductivity (∼150 mS m^-1^), while the composite hydrogel prepared with 0.5 wt% PB maintained moderate and stable conductivity (∼25 mS m^-1^) throughout 14 days (Figure 1g). Notably, all electrical characterizations in this study were conducted at the optimized and biologically safe PB loading (0.5 wt%), as determined by cytocompatibility screening (Busquets and Estelrich, 2020). Unless otherwise specified, all physicochemical, electrical, and biological characterizations in this study were performed using PBM. PVA hydrogel containing 0.5 wt% PB, which was selected based on cytocompatibility screening and IC_10_ analysis (Figures 1i,j). While IC_10_ evaluation was used to define the optimal material composition, extract-based cytocompatibility assays were further conducted to assess the safety of the final PBM. PVA hydrogel formulation under practical usage conditions (Supplementary Figure S2). Diabetic wounds typically lose their natural trans-epithelial potential (TEP) and exhibit greatly weakened wound-associated DC electric fields that normally provide directional cues for keratinocyte migration and angiogenesis. The electroactive and conductive nature of PBM. PVA hydrogel may help sustain a permissive electrical microenvironment and facilitate the transmission of endogenous bioelectric cues in diabetic wounds, without implying that the material actively generates such signals (Ullah et al., 2023), allowing keratinocytes and endothelial cells to re-orient and respond to subtle DC field cues even without external stimulation. Endogenous wound bioelectric cues originate from disruption of transepithelial potential and ionic gradients, and have been reported as lateral electric fields of ∼40–200 mV/mm with injury currents in the µA·cm^-2^ range. This phenomenon suggests that the hydrogel may provide a permissive electrical microenvironment that supports cell orientation and migration in diabetic wounds, without implying a defined mechanistic pathway. In addition to its excellent adhesion and hemocompatibility, the injectability of the PBM. PVA hydrogel was quantitatively evaluated by rheological analysis. As shown in Figure 2g, the viscosity of the PBM. PVA hydrogel decreased markedly with increasing shear rate, exhibiting a typical shear-thinning (pseudoplastic) behavior. The apparent viscosity dropped by more than one order of magnitude as the shear rate increased from low to high values, indicating that the hydrogel can readily flow under shear stress during injection while maintaining structural integrity at rest. This pronounced shear-thinning behavior is highly desirable for injectable biomaterials, as it enables smooth extrusion through a syringe needle with low resistance while preventing leakage or spreading after administration. These results provide quantitative rheological evidence supporting the excellent injectability of the PBM. PVA hydrogel. Strong interfacial adhesion was maintained across various substrates, including human skin, biological tissues, and multiple synthetic surfaces, due to hydrogen bonding and polymer chain entanglement at the hydrogel-tissue interface (show as Figure 2a). Quantitative lap-shear measurements demonstrated that the PBM. PVA hydrogel exhibited strong and substrate-dependent adhesion across a wide range of materials. Specifically, the lap-shear strength reached 55 ± 8 kPa on glass, 45 ± 7 kPa on ceramics, 40 ± 6 kPa on aluminum alloy, 35 ± 6 kPa on iron/steel, 22 ± 5 kPa on wood, 18 ± 4 kPa on rubber, 4 ± 1 kPa on polyethylene (PE), 12 ± 3 kPa on beef tissue, and 8 ± 2 kPa on shrimp tissue, respectively (Figure 2d). All values are presented as mean ± SD (n = 5). Hemolysis assays confirmed <1% hemolysis, showing excellent blood compatibility (Figures 2b,e). Statistical analysis has been included in the graph to further support these findings, particularly in the context of diabetic wounds where vascular fragility and bleeding risk are elevated. Furthermore, FITC-labeled PBM. PVA gel exhibited stable fluorescence retention following intradermal injection (Figure 2c), confirming effective *in situ* gelation and reliable localization properties crucial for deep or irregular diabetic wound beds (Zhang et al., 2023; Wu et al., 2025; Yan et al., 2025). As shown in the frequency sweep results (Figure 2f), the storage modulus (G′) remained consistently higher than the loss modulus (G″) across the entire frequency range without any crossover, indicating a stable elastic-dominant network and good resistance to structural disruption under dynamic mechanical loading. This temperature-controlled rheology at 37 °C addresses the concern that freeze-thaw PVA hydrogels may soften near body temperature, and indicates that the present PBM. PVA formulation maintains a stable elastic network under physiological conditions within the tested timeframe.

As shown in Figures 4a–c, PBM. PVA treatment significantly accelerated wound closure compared with the control, PVA, and PBM groups. The wounds treated with PBM. PVA gel exhibited visibly smaller residual areas from day 3 onward, indicating an earlier onset of re-epithelialization. Quantitative analysis confirmed that the PBM. PVA group showed the fastest healing kinetics, achieving more than 80% closure by day 7, whereas the control and PVA groups displayed only limited contraction within the same period. The enhanced healing progression observed in the PBM. PVA group is associated with improved wound closure and reduced inflammatory marker expression at the optimized PB loading. In this study, we limit our interpretation to the observed biological outcomes and do not assign a defined mechanistic contribution to electroactive or antioxidative features. Notably, the PBM-only group showed partial improvement but remained inferior to PBM. PVA gel, underscoring the synergistic role of the hydrated PVA matrix in supporting cellular infiltration and maintaining a moist repair microenvironment. In LPS-activated macrophages, we used 1 μg/mL LPS, a commonly accepted dose in macrophage activation studies, which has been extensively validated in our previous works (Chang and Cao, 2021).

**FIGURE 3:**
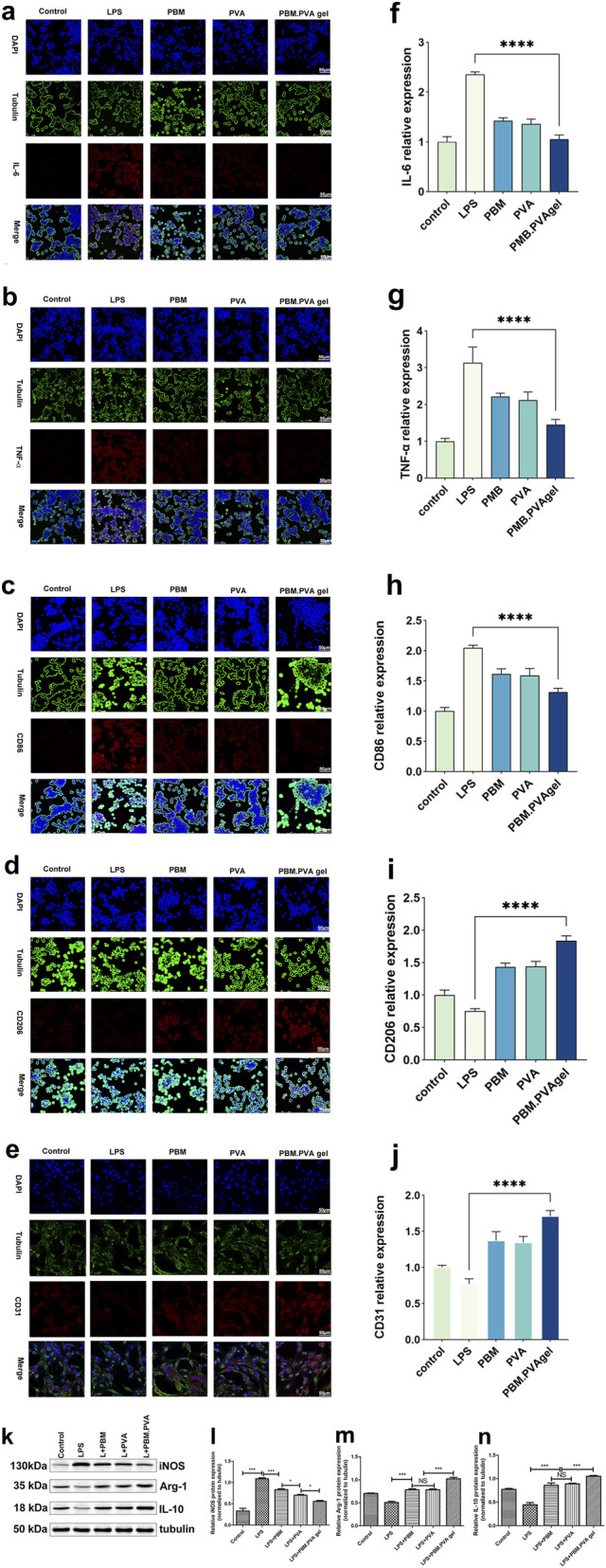
*In vitro* immunomodulatory and endothelial-related responses to PBM. PVA hydrogels. **(a–c)** Immunofluorescence staining of IL-6, TNF-α, and CD86 in RAW264.7 macrophages. **(d–e)** Immunofluorescence staining of CD206 in RAW264.7 macrophages and CD31 in HUVECs. **(f–j)** Quantitative analysis of fluorescence intensity corresponding to the immunofluorescence images in panels **(a–e)**. **(k–n)** Western blot analysis and corresponding densitometric quantification of iNOS, Arg-1, and IL-10 protein expression levels in LPS-stimulated cells treated with PBM, PVA, or PBM. PVA gel, with tubulin used as the internal loading control. Data are presented as mean ± SD (n = 3).

The PBM. PVA hydrogel markedly reduced the expression of IL-6, TNF-α, CD86 and iNOS, while simultaneously increasing CD206, Arg-1, IL-10 and CD31 levels (Figures 3a–n, 6). These changes indicate an effective suppression of pro-inflammatory M1 activity and a shift toward a pro-regenerative M2 phenotype, reflecting the hydrogel’s ability to mitigate excessive inflammation in the diabetic wound microenvironment (Yang et al., 2025).

**FIGURE 4:**
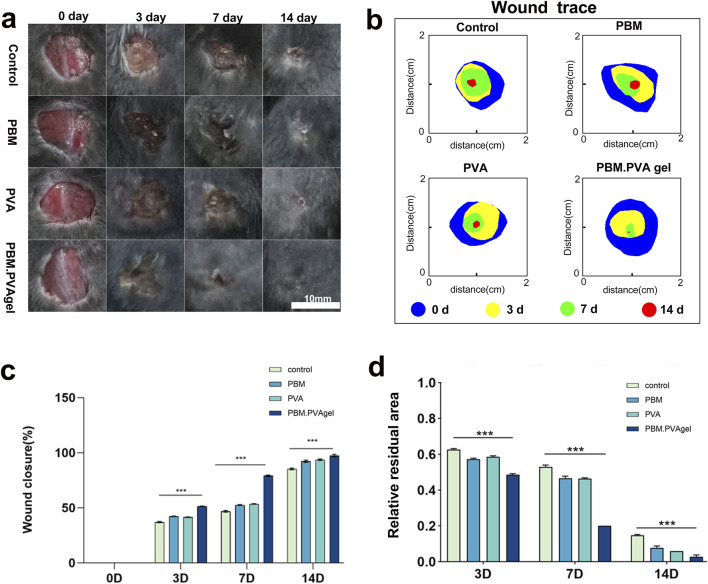
*In vivo* wound healing and scar-related outcomes in diabetic mice. **(a)** Representative photographs of wounds at 0, 3, 7, and 14 days after treatment in the Control, PBM, PVA, and PBM. PVA gel groups (scale bar = 10 mm). **(b)** Wound trace overlays showing the temporal reduction of wound area at 0 (blue), 3 (yellow), 7 (green), and 14 (red) days for each group. **(c)** Quantification of wound closure (%) at days 0, 3, 7, and 14. **(d)** Quantification of relative residual area at days 3, 7, and 14. Data are presented as mean ± SD (n = 15). Statistical significance is indicated in the figure (***P < 0.001).

**FIGURE 5:**
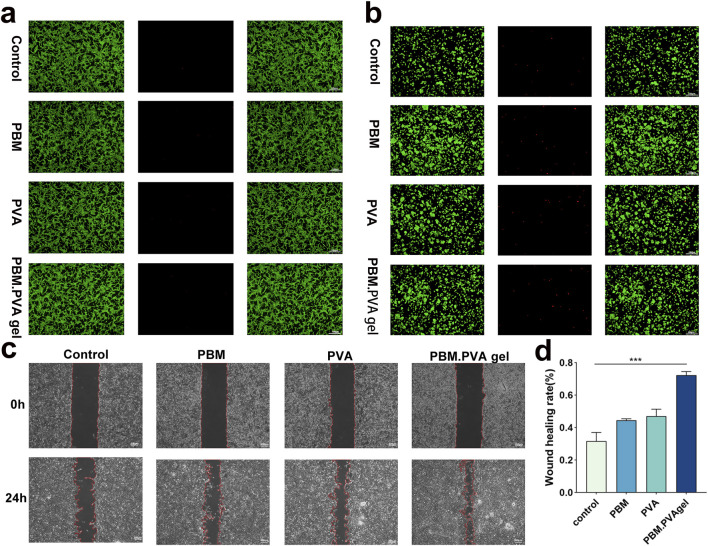
*In vitro* cytocompatibility and migration behavior of PBM. PVA hydrogels. **(a–b)** Live/dead staining of RAW264.7 macrophages and HUVECs after 48 h with PBM. PVA gel (green: live cells; red: dead cells). **(c)** Representative scratch wound images of HUVECs at 0 and 24 h. **(d)** Quantitative analysis of cell migration showing significantly enhanced wound closure in the PBM. PVA group. Data are presented as mean ± SD (n = 3).

The biocompatibility of the PBM. PVA hydrogel was further evaluated in a concentration-dependent manner using extract dilution assays. As shown in Supplementary Figure S2, both RAW264.7 macrophages and HUVECs maintained high viability (>90%) at extract concentrations up to 75%, with only a slight decrease observed at 100% extract, confirming good cytocompatibility within the practical usage range. These results provide supplementary support for the excellent biocompatibility observed in Figures 4a,b. In scratch assays, PBM. PVA hydrogel promoted markedly enhanced HUVEC migration, achieving approximately a 2.1-fold increase compared with the control group (Figures 5c,d), indicating an early pro-angiogenic endothelial response. This improved migratory performance suggests that the hydrogel provides a favorable microenvironment for endothelial activation and early angiogenic responses, consistent with its antioxidant capacity and hydrated matrix features (Zhou et al., 2026).

**FIGURE 6:**
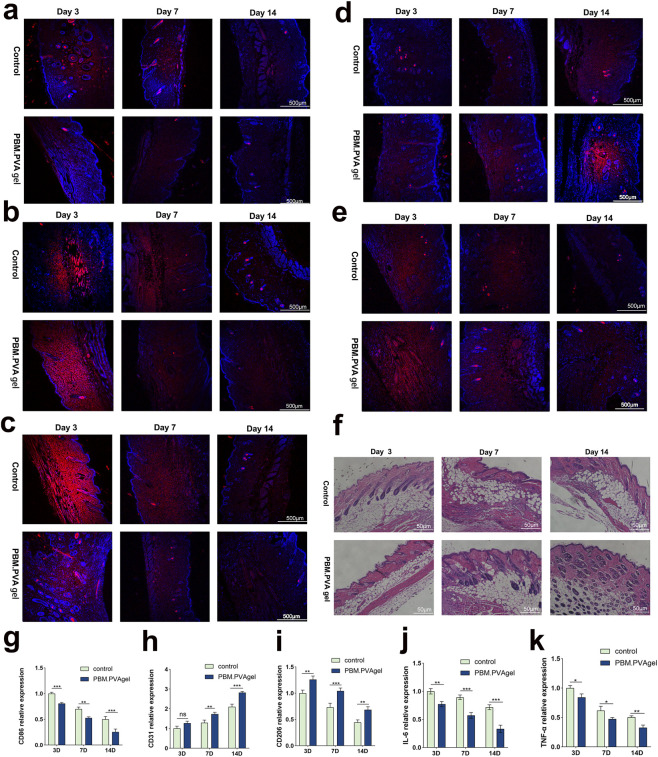
Histological and immunofluorescence analysis of wound tissues. **(a–e)** Immunostaining of IL-6, TNF-α, CD86, CD31, and CD206 in wound sections. **(f)** H&E staining of wound tissues showing epithelial regeneration and collagen deposition. **(g–k)** Quantitative analysis of fluorescence intensity of inflammatory and angiogenic markers at different time points. Data are presented as mean ± SD. Author notes: The graphical abstract is provided as a standalone item and is not included in the main figure numbering. All figures have been renumbered and arranged to strictly follow their first citation in the text, and the corresponding legends have been revised for clarity and consistency.

The therapeutic efficacy of the PBM. PVA hydrogel was further evaluated in an STZ-induced diabetic mouse model. As shown in Figure 6f, histological staining revealed that wounds treated with PBM. PVA hydrogel exhibited markedly denser and more orderly aligned collagen fibers, along with a more complete and stratified epidermal architecture at day 14, reflecting enhanced early-stage matrix deposition and re-epithelialization rather than mature collagen remodeling or scar formation, compared with the control groups. These findings indicate that the hydrogel facilitates granulation tissue formation and early tissue regeneration, thereby establishing a favorable microenvironment for subsequent wound healing.

Notably, this pronounced enhancement in tissue repair is consistent with the findings reported by Yang et al., He et al., and Kang et al., who demonstrated that multifunctional hydrogels can significantly promote structural remodeling and accelerate wound regeneration in diabetic models (Yang et al., 2024; He et al., 2025; Karthikeyan and Kang, 2025). These parallels further support the efficacy of the PBM. PVA hydrogel in modulating inflammation, enhancing matrix deposition, and expediting wound closure.

Chronic diabetic wounds represent one of the most complex and therapeutically refractory forms of tissue injury, largely due to the convergence of three pathological hallmarks: (i) elevated oxidative stress, (ii) persistent inflammation, and (iii) the often overlooked but fundamentally impaired endogenous bioelectric microenvironment. Under physiological conditions, intact skin maintains a trans-epithelial potential (TEP) that generates a directional DC electric field upon injury, guiding keratinocyte migration, orchestrating angiogenesis, and initiating early wound activation. However, in diabetic wounds, these bioelectric gradient collapses, leaving the wound bed electrically silent and biologically unresponsive, which significantly compromises tissue regeneration (Mai et al., 2025). This intrinsic deficit highlights the need for a dressing capable not only of modulating biochemical imbalances but also of reinstating functional bioelectric cues (Liu et al., 2020; Bikmulina et al., 2022). Within this context, we designed the PBM. PVA composite hydrogel to address these multifactorial deficits. The electrospun Prussian Blue nanofibers form an interconnected conductive network that enables continuous electron/ion transport; however, endogenous wound electric fields were not directly measured in this study. Therefore, we describe the PBM. PVA hydrogel as a passive electroconductive matrix that may facilitate ionic/electronic transport in the wound environment, rather than claiming reconstruction of DC field propagation. Unlike conductive systems that rely on external power sources, the PBM. PVA hydrogel is designed as a passive electroactive matrix that can facilitate ionic/electronic transport and potentially support the transmission of endogenous bioelectric cues in the wound microenvironment, without applying external stimulation. This intrinsic electroactivity provides a unique advantage in diabetic settings, although electrical cues have been reported to influence cell behavior in wound repair, we did not perform electrophysiological mapping or calcium signaling assays in this study. Accordingly, we limit our interpretation to the observed biological outcomes, including enhanced HUVEC migration in scratch assays and increased CD31-related signals, without assigning a defined electrical signaling mechanism (Lu et al., 2025; Zhang et al., 2025). Beyond its electrical functionality, PBM has been reported to possess redox-regulatory potential due to its mixed-valence Fe^2+^/Fe^3+^ with reversible redox behavior, which may contribute to redox regulation in biological microenvironments. However, in this study, we did not directly characterize Fe valence transitions within the PBM. PVA gel using CV or XPS (Su et al., 2025). Therefore, we discuss Fe^2+^/Fe^3+^ redox cycling as a plausible, literature-supported mechanism, rather than a mechanism experimentally confirmed in our gel system.

**SCHEME 1:**
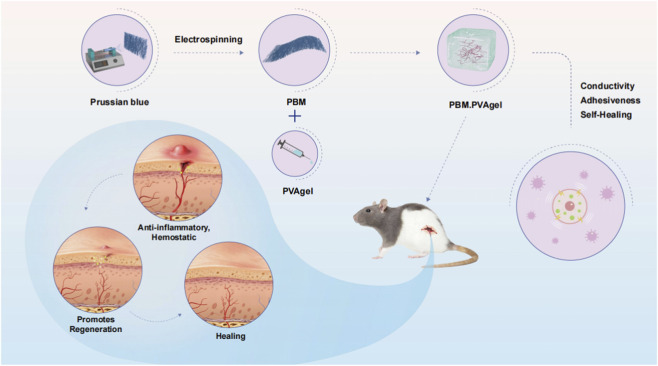
Schematic illustration of the wound-healing mechanism mediated by PBM. PVA hydrogel. An injectable PBM. PVA hydrogel restores the impaired bioelectric microenvironment of diabetic wounds through a self-conductive PB nanofiber network that transduces endogenous DC electric fields. Together with potential antioxidant-related and immunomodulation, the hydrogel promotes directional cell migration, angiogenesis, and rapid re-epithelialization, enabling efficient regeneration of chronic diabetic wounds without external electrical stimulation.

In this work, we did not directly quantify intracellular ROS levels using assays such as DCFH-DA staining or EPR. Therefore, we describe PBM. PVA as having redox-regulatory potential based on prior reports and the observed attenuation of inflammation-related signaling, rather than claiming definitive ROS scavenging confirmation. In the present study, PB incorporation at 0.5 wt% is intended to leverage this reported antioxidant potential while maintaining cytocompatibility. This reported redox-regulatory potential may contribute to the observed attenuation of pro-inflammatory signaling (IL-6, TNF-α, and CD86) and the promotion of CD206^+^ M2 macrophage polarization-shifting the inflammatory milieu from a destructive to a pro-regenerative state (Sahu et al., 2021; Xu et al., 2022; Zhou et al., 2026). Simultaneously, the hydrated and compliant PVA matrix provides a favorable microenvironment for endothelial function, and in combination with PBM’s bioelectrical and redox activities, significantly enhances angiogenic signaling. The marked increase in CD31^+^ neovascular structures aligns with the well-documented role of electrical and redox cues in stimulating endothelial sprouting and maturation. Thus, the enhanced CD31-positive signals observed *in vivo* are associated with a wound environment characterized by reduced inflammatory markers and improved tissue repair outcomes (Su et al., 2019; Guan et al., 2021; Wang et al., 2024; Zhao et al., 2025). Because ROS levels and bioelectric parameters were not directly quantified, we do not infer a causal ROS-bioelectric-angiogenesis cascade; these relationships remain to be clarified in future mechanistic studies. Importantly, the PBM. PVA hydrogel treatment is associated with improved wound closure, enhanced collagen deposition, and reduced inflammatory marker expression in diabetic mice. These outcomes collectively indicate improved wound healing performance within the scope of the present experimental observations. Compared with existing hydrogel dressings that typically target single pathological factors, the PBM. PVA system integrates electroactive, antioxidative, and immunomodulatory features at the material level, which together may contribute to the observed improvement in wound healing outcomes (Xiang et al., 2024; Yang et al., 2024; Altalbawy et al., 2025). The proposed multifunctional mechanism by which PBM. PVA modulates the diabetic wound microenvironment is summarized in Scheme 1.

A limitation of this study is that a Matrigel-based tube formation assay was not performed to evaluate capillary-like network formation *in vitro*. Future work will incorporate tube formation assays to provide a more comprehensive *in vitro* angiogenesis assessment. It should be noted that the present study primarily focused on the early to intermediate stages of diabetic wound healing, with evaluation endpoints up to 14 days, including wound closure, granulation tissue formation, re-epithelialization, and early collagen matrix deposition. Although improved collagen density and organization were observed at day 14, these findings represent early extracellular matrix deposition rather than mature collagen remodeling or definitive scar formation. Late-stage collagen alignment, fiber reorganization, and scar maturation processes typically occur over longer time periods and were not directly assessed in this study. Therefore, no claims are made regarding long-term scar reduction or remodeling outcomes. Future studies extending the observation period will be necessary to systematically evaluate collagen alignment, scar architecture, and functional tissue remodeling at later stages of healing.

Despite the encouraging therapeutic outcomes observed in this study, several aspects warrant further investigation. Future work will focus on a more comprehensive characterization of the PBM. PVA gel, including its degradation behavior and redox-related physicochemical properties, as well as deeper exploration of its bioelectrical and oxidative stress modulation effects. Although the PBM. PVA hydrogel exhibits stable electrical conductivity and is discussed as a passive electroactive matrix that may support endogenous bioelectric cue transmission, direct quantification of trans-epithelial potentials (TEP) and endogenous wound currents was not performed in this study. In future work, we will incorporate electrophysiological measurements to more directly interrogate the bioelectric microenvironment. Specifically, barrier integrity and ion transport will be assessed using TEER/TEP measurements in relevant epithelial/endothelial monolayer models (e.g., keratinocyte or endothelial barrier systems). In parallel, microelectrode- or vibrating-probe-based mapping will be used to quantify injury currents and local electric field distributions at wound margins in diabetic models. In addition, long-term tissue remodeling outcomes, such as collagen organization and scar formation, will be evaluated to further assess the quality and durability of wound healing. These studies will help to provide a more complete understanding of the long-term performance and translational potential of this system. Moreover, future work will include systematic benchmarking against representative commercial hydrogel dressings and established conductive hydrogel formulations to better contextualize the therapeutic performance of PBM. PVA. In parallel, PB nanoparticle-only controls will be evaluated using matched PB dosing and standardized administration/retention conditions to enable a fair comparison with the PBM-based fibrous network embedded in the hydrogel.

In summary, the PBM. PVA hydrogel is a conductive and immunomodulatory dressing that improves diabetic wound healing outcomes *in vivo* and reduces inflammatory marker expression *in vitro*. The present conclusions are strictly based on the experimental evidence obtained in this study. By providing an electroactive and redox-modulating microenvironment, the PBM. PVA hydrogel supports cellular responses associated with improved wound closure and tissue regeneration in diabetic mice.

## 4 Conclusion

An injectable, adhesive, and biocompatible Prussian Blue nanofiber-PVA hydrogel was developed for diabetic wound healing applications. The PBM. PVA gel demonstrated superior mechanical performance, *in vitro* immunomodulatory effects (reduced pro-inflammatory markers and increased pro-regenerative markers), and enhanced endothelial-related responses (HUVEC migration and increased CD31-related signals), together with improved wound closure and tissue repair *in vivo*. This composite hydrogel provides a promising strategy for the management of chronic diabetic ulcers and related tissue repair disorders.

## Data Availability

The original contributions presented in the study are included in the article/Supplementary Material, further inquiries can be directed to the corresponding author.
