# Correction: Injectable electroconductive Prussian blue nanofiber-PVA hydrogel modulates the wound microenvironment to promote diabetic wound healing

**DOI:** 10.3389/fbioe.2026.1844032

**Published:** 2026-04-21

**Authors:** Dian-Qing Wang, Jin Huang, Sheng Chang

**Affiliations:** 1 Department of Orthopedics, The First Affiliated Hospital of Jinzhou Medical University, Jinzhou, Liaoning, China; 2 Key Surgical Laboratory of Educational Administration of Liaoning Province, The First Affiliated Hospital of Jinzhou Medical University, Jinzhou, Liaoning, China; 3 Department of Stomatology, The First Affiliated Hospital of Jinzhou Medical University, Jinzhou, Liaoning, China

**Keywords:** diabetic wound healing, electroactive biomaterial, hydrogel, injectable hydrogel, Prussian blue nanofiber

Author Jin Huang was erroneously omitted as corresponding author. The correct Corresponding authors are Jin Huang and Sheng Chang.

The original article has been updated.

