# Supplementary figures and images for: Injectable electroconductive Prussian blue nanofiber-PVA hydrogel modulates the wound microenvironment to promote diabetic wound healing
Source: Front Bioeng Biotechnol. 2026 Feb 16;14:1748784. doi: 10.3389/fbioe.2026.1748784 (PMC12950806; doi:10.3389/fbioe.2026.1748784)

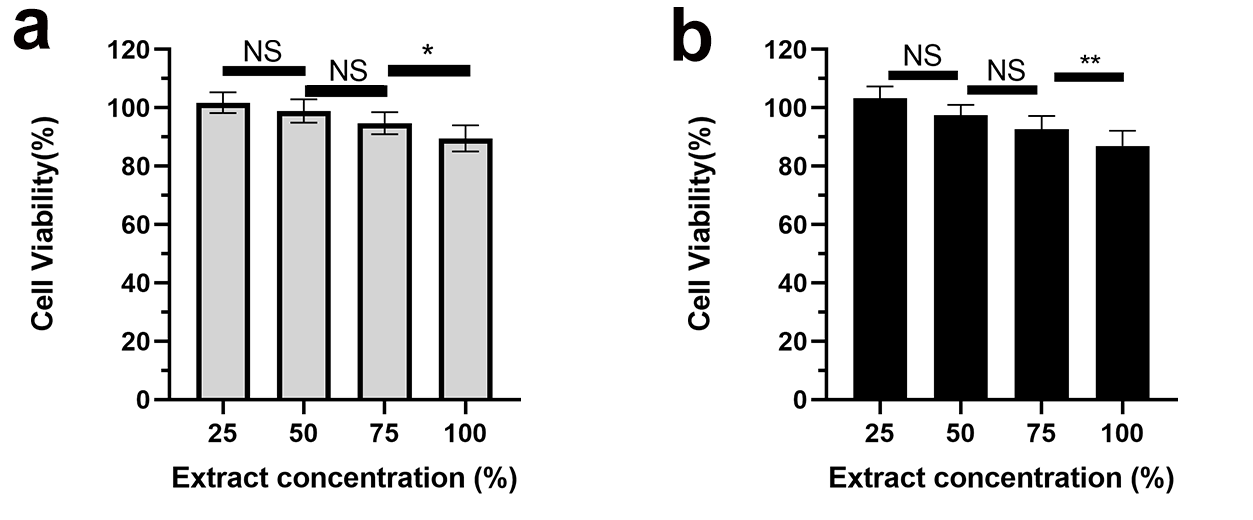

Supplement: Supplementary file 2 [file Image2.tif]

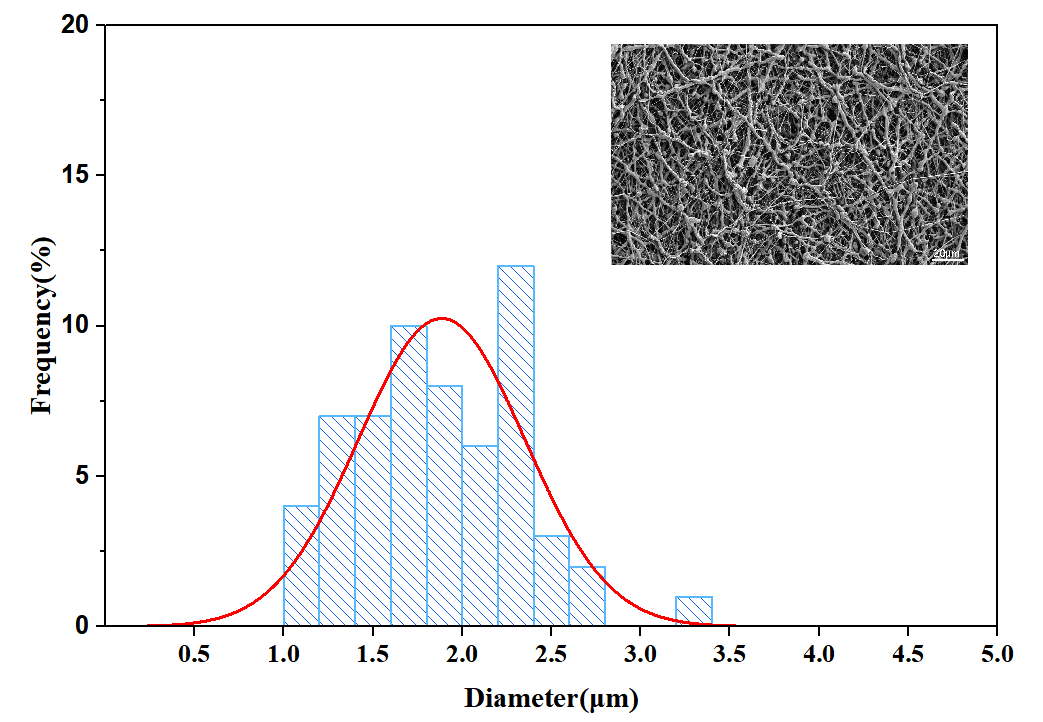

Supplement: Supplementary file 3 [file Image1.tif]
